# A novel and high-efficient method for the preparation of heat-stable antifungal factor from *Lysobacter enzymogenes* by high-speed counter-current chromatography

**DOI:** 10.3389/fmicb.2023.1227244

**Published:** 2023-08-14

**Authors:** Weibo Sun, Bao Tang, Liangliang Dong, Jianhong Xu, Yancun Zhao, Fengquan Liu

**Affiliations:** ^1^Jiangsu Key Laboratory for Food Quality and Safety-State Key Laboratory Cultivation Base of Ministry of Science and Technology, Institute of Plant Protection, Jiangsu Academy of Agricultural Sciences, Nanjing, China; ^2^College of Plant Protection, Anhui Agricultural University, Hefei, China; ^3^Institute of Food Safety and Nutrition, Jiangsu Academy of Agricultural Sciences, Nanjing, China

**Keywords:** heat-stable antifungal factor, *Lysobacter enzymogenes*, separation, preparation, high-speed counter-current chromatography

## Abstract

Heat-stable antifungal factor (HSAF) produced by the biocontrol bacterium *Lysobacter enzymogenes* shows considerable antifungal activity and has broad application potential in the agricultural and medical fields. There is a great demand for pure HSAF compounds in academic or industrial studies. However, an efficient preparation method that produces a high yield and high purity of HSAF is lacking, limiting the development of HSAF as a new drug. In the present study, high-speed counter-current chromatography (HSCCC) combined with column chromatography was successfully developed for the separation and preparation of HSAF from the crude extract of *L. enzymogenes* OH11. The crude extract was obtained by macroporous resin adsorption and desorption, and the main impurities were partly removed by ultraviolet light (254 nm) and gel filtration (Sephadex LH-20). In the HSCCC procedure, the selected suitable two-phase solvent system (*n*-hexane/ethyl acetate/methanol/water = 3:5:4:5, v/v, the lower phase added with 0.1% TFA) with a flow rate of 2.0 mL/min and a sample loading size of 100 mg was optimized for the separation. As a result, a total of 42 mg HSAF with a purity of 97.6% and recovery of 91.7% was yielded in one separation. The structure elucidation based on HR-TOF-MS, ^1^H and ^13^C NMR, and antifungal activities revealed that the isolated compound was unambiguously identified as HSAF. These results are helpful for separating and producing HSAF at an industrial scale, and they further demonstrate that HSCCC is a useful tool for isolating bioactive constituents from beneficial microorganisms.

## 1. Introduction

Plant diseases caused by various pathogens have caused great losses in the yield and quality of agricultural products worldwide. Biological control technology uses beneficial living microorganisms or their secondary metabolites to inhibit the growth and spread of plant pathogens, which is conducive to agricultural and ecological health. For a long time, using natural products derived from beneficial microbes to control plant diseases attracted extensive attention among scientists.

**Figure 1:**
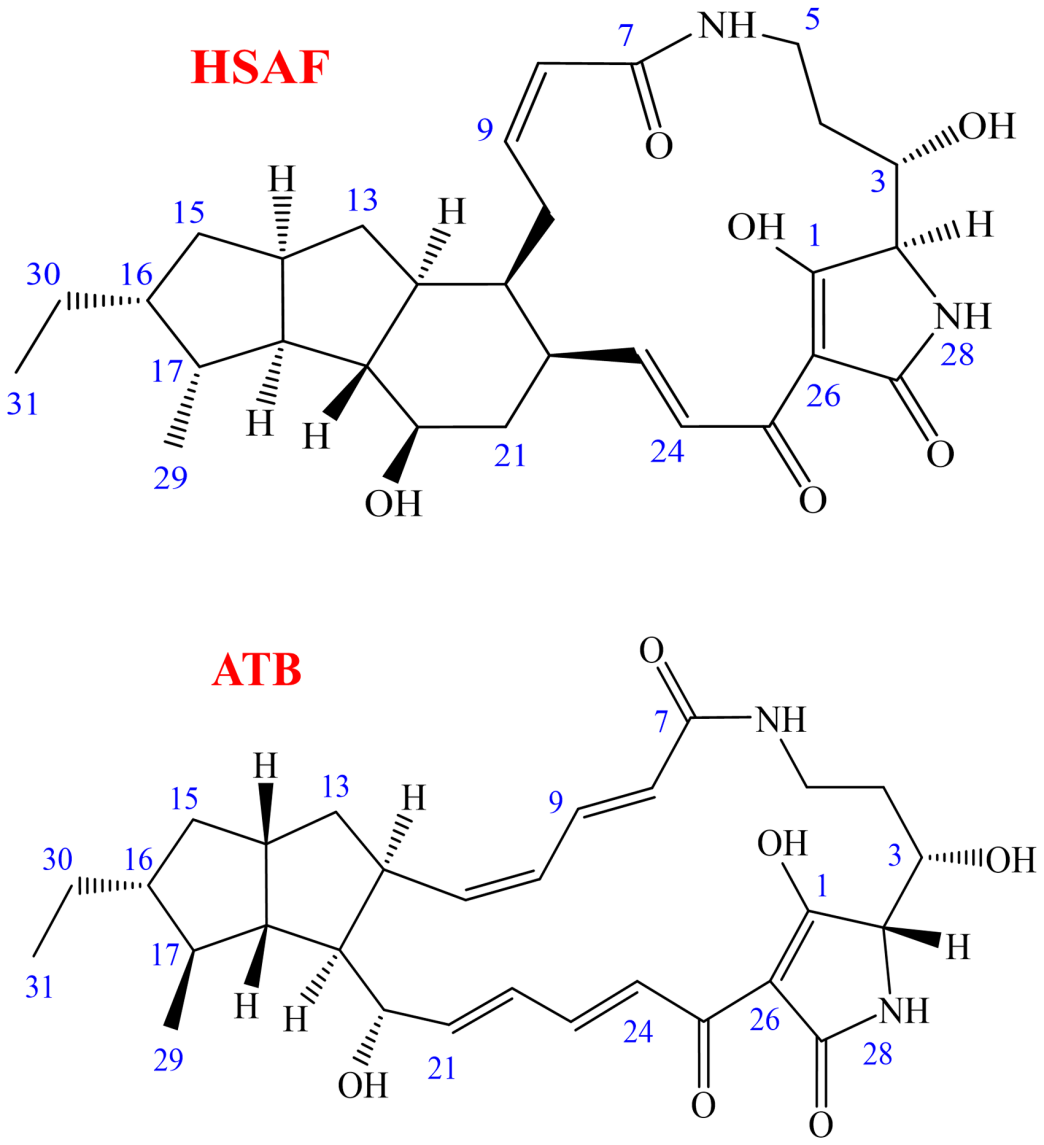
Chemical structures of HSAF (dihydromaltophilin) and ATB (alteramide B).

In our continuous search for valuable natural products derived from microbes to control plant diseases, we found that the compound heat-stable antifungal factor (HSAF), also known as dihydromaltophilin, produced by *Lysobacter enzymogenes* showed strong antagonistic activities against various plant fungal diseases. *L. enzymogenes*, which was first described in 1978, belongs to the *Xanthomodaceae* family and has emerged as a potential biocontrol agent in the suppression of fungal and oomycete diseases (Christensen and Cook, 1978; Zhao et al., 2017; Lin et al., 2021). HSAF, which was originally isolated from a *Streptomyces* sp. strain in 1997, is an antimicrobial secondary metabolite produced by *L. enzymogenes* strains such as C3, C3R5, YC36, and OH11 (Graupner et al., 1997; Xu et al., 2015; Zhao et al., 2017; Xu et al., 2018; Zhong et al., 2022). HSAF is a polycyclic tetramate macrolactam compound and its chemical structure contains a tetramic acid moiety and a 5/5/6-tricyclic skeleton, which is different from the structure of any existing antifungal drug (Figure 1) (Li et al., 2006, 2009, 2012; Xu et al., 2015). HSAF shows antagonistic activity against fungi and oomycetes by disrupting sphingolipid biosynthesis, and it can induce the apoptosis of *Candida albicans* cells by promoting the accumulation of reactive oxygen species (ROS) (Li et al., 2009; Ding et al., 2016a,b). In agricultural fields, we have proven that HSAF could be used as a new natural defender against plant diseases, as it can effectively prevent Fusarium head blight in wheat caused by *Fusarium graminearum* (Zhao et al., 2019), and it can also effectively protect pear fruits from anthracnose caused by *Colletotrichum fructicola* (Li et al., 2021; Zhao et al., 2021). These results demonstrate its promise in the development of biological control agents. Furthermore, many macrolactam compounds showed remarkable pharmaceutical activities (Shin et al., 2023), and HSAF also showed promise to become a new drug in the medical field, as the latest study has shown that the Collagen-HSAF has the potential to be developed as an antifungal drug with significant clinical value in the treatment of superficial fungal infections in humans (Zhong et al., 2022).

However, developing HSAF as a new commercialized biocontrol agent or medical drug requires a large amount of work on both the biological and chemical side, including toxicological studies, risk assessment, registration procedure, and mechanism of action studies. All of these requirements will consume large amounts of the pure compound, increasing the demand for pure HSAF compounds. At present, the total synthesis of HSAF has not yet been achieved, and the only source of HSAF is the fermentation broth of microorganisms (Zhong et al., 2022). Unfortunately, many analogous compounds such as alteramides and lysobacteramides exist in the crude extracts (Li et al., 2012; Xu et al., 2015; Ding et al., 2016a,b), and because they share a common structural scaffold and differ mainly at the fused cyclic system (Li et al., 2018), it is difficult to isolate HSAF as a pure compound. Traditional separation and purification methods, including column chromatography, macroporous resin adsorption/desorption, and preparative high-performance liquid chromatography (prep-HPLC) were often used in the previous studies (Graupner et al., 1997; Xu et al., 2015; Tang et al., 2021; Zhong et al., 2022). All of these methods were based on solid–liquid adsorption and there was a large amount of sample loss due to irreversible adsorption. The yield of pure product was low and the recovery was not good, so the separation procedure was at low efficiency. In addition, the high cost of the HPLC instruments and solvents makes its use difficult in large-scale production. Based on this, it is urgently desirable to develop an efficient and powerful technology for the preparation of HSAF.

High-speed counter-current chromatography (HSCCC) is an advanced form of support-free liquid–liquid partition chromatography (Ito, 2005). It relies on the repeated partition of samples between the two phases of an immiscible solvent system (Yao et al., 2012). The compounds are separated according to their different partition coefficients (K value). The two phases (upper and lower phase) are used as the stationary and mobile phase with no solid support matrix, thus, the problem of irreversible adsorption of samples is eliminated and the recovery of the samples can theoretically reach nearly 100%. The fractions can be collected based on the chromatograms, and the purities of the products can be above 95% (He et al., 2013; Kang et al., 2016; Yuan et al., 2020). As a result, these advantages made HSCCC widely used for the separation of bioactive components including antibiotics, antioxidants, enantiomers, and medicines from plants, foods, or beneficial microorganisms (Oka et al., 1998; Sutherland and Fisher, 2009; Huang et al., 2018; Gong et al., 2020). More importantly, HSCCC is easily expanded to industrial-scale production if there is appropriate commercial support. For example, it was reported that 23.6 g of glucoraphanin or 20 g of honokiol with purities >98% could be yielded in one injection of HSCCC (Sutherland, 2007), thus the two valuable compounds were easily produced in the factory. Furthermore, manufacturers in Shanghai or Jiangyin, China, developed the preparative HSCCC instruments with a column capacity that reached 5 L or 30 L, allowing the production of target compounds to reach the 10 g or 100 g level. The rapid progress of HSCCC technology has aroused our strong interest in the development of new drugs.

However, there are very few reports on the use of HSCCC to isolate compounds from the fermentation broth of *Lysobacter* sp., except for the isolation of the WAP-8294A complex in 2001 (Harada et al., 2001). Using HSCCC to separate HSAF was also unreported. In this study, HSCCC was used for the separation and preparation of the antifungal component HSAF from the *L. enzymogenes* crude extract. The partition coefficients for HSAF and the main interferent in different solvent systems were measured and the separation conditions were optimized. The structure and antifungal activities of the product were verified by spectroscopic experiments and biological tests. To the best of our knowledge, this is the first study to use HSCCC to separate macrolactam compounds from *Lysobacter* sp. Our results showed that HSCCC is a powerful tool for the separation and preparation of bioactive compounds from natural microorganisms.

## 2. Materials and methods

### 2.1. Apparatus

The HSCCC instrument was an OptiChrome-300 PLUS high-speed counter-current chromatograph system (Counter-current Technology, Jiangyin, China). The apparatus had a rotational speed of 400–1,200 rpm, with a centrifugal force of up to 110 g, and was equipped with two polytetrafluoroethylene (PTFE) multilayer coils (210 m × 1.6 mm id) with a total capacity of 300 mL and a 30 mL manual sample loop (Wang et al., 2019). The solvent was pumped into the column with a *CF*-80 pump (Counter-current Technology, Jiangyin, China). The effluent was detected at 318 nm with a UV2000D ultraviolet detector (Sanotac Scientific Instruments, Shanghai, China). An EasyChrom-1,000 chromatography workstation (Hanbon Science and Technology, Huaian, China) was used to record the chromatogram.

Analytical HPLC was performed using a Shimadzu LC-20A system (Shimadzu, Kyoto, Japan) equipped with two Shimadzu 6 AD pumps, an analytical Agilent ZORBAX SB-C18 column (4.6 × 250 mm, 5 μm), and an SPD-M20A diode array detector with UV detection at wavelengths of 190–500 nm. High-resolution time-of-flight mass spectrometry (HR-TOF-MS) for the isolated compound was performed using a Triple TOF 5600+ instrument (AB SCIEX, America). The nuclear magnetic resonance (NMR) spectra ^1^H NMR and ^13^C NMR were recorded using a BRUKER AVANCE 3 instrument at 400 MHz and 100 MHz, respectively. The compound was dissolved in dimethyl sulfoxide-*d*_6_ (DMSO-*d*_6_) with 0.03% tetramethylsilane (TMS) as the inner standard.

### 2.2. Reagents and microorganisms

All organic solvents used for the preparation of crude extract, column chromatography, and HSCCC separation were of analytical grade (Sinopharm Chemical Reagent, Shanghai, China). For HPLC, the mobile phase acetonitrile was HPLC grade (TEDIA, Anhui, China) and the water was purified using a Milli-Q water purification system (Millipore, United States).

The bacterium strain *L. enzymogenes* OH11 used in this study was originally isolated from the rhizosphere of a green pepper plant in our laboratory and deposited at the Center of General Microbiological Culture Collection (CGMCC No. 1978) in China (Tang et al., 2018a,b; Tang et al., 2019). The plant pathogens used in the bioactivity assay, including *Fusarium graminearum*, *Colletotrichum fructicola*, *Alternaria alternata*, *Botryosphaeria dothidea*, and *Valsa pyri*, were preserved in our laboratory (Zhao et al., 2019; Li et al., 2021).

### 2.3. Fermentation and preparation of HSAF crude extract

The HSAF crude extract was prepared using the selected fermentation conditions and the macroporous resin adsorption/desorption method according to our previous study (Tang et al., 2018a,b; Tang et al., 2021). Briefly, the *L. enzymogenes* OH11 strain activated on a solid Luria-Bertani (LB) medium was inoculated into an LB liquid medium and incubated at 28°C for 12 h with shaking at 180 rpm as the seed culture. Then, the seed culture was inoculated (2% v/v) into the defined SGC medium (8.00 g/L soybean flour, 7.89 g/L glucose, and 0.72 g/L calcium ion) and incubated for 60 h. Next, the macroporous resin NKA (15 g/L) was added into the fermentation broth and the culture resumed shaking for another 12 h, and most of the HSAF produced in the broth was adsorbed into the resin. The sample solutions were filtered using gauze, and the resin was washed with ethanol (shaken for 2 h and repeated three times) to desorb HSAF.

To improve the HSAF resolution in the following separation procedure, the desorption solution was pretreated to remove impurities. The ethanol desorption eluent was placed under an ultraviolet germicidal lamp (UV wavelength at 254 nm) for 6 h, then it was concentrated using a rotary evaporator. The brown gum of HSAF extract was subjected to Sephadex LH-20 gel column chromatography and eluted with dichloromethane-methanol = 1:1. The fractions containing HSAF were combined and evaporated to dryness.

### 2.4. Selection of two-phase solvent system in HSCCC

The selection of a suitable immiscible two-phase solvent system for the target compound is the most important step in HSCCC, and it is estimated to account for approximately 90% of the entire work (Ito, 2005). In general, the two-phase solvent system was selected according to the partition coefficient (K value) of the target component. The K value for the target compound was determined as follows: a small amount of crude sample (10 mg) was added into a test tube containing approximately 20 mL of a two-phase solvent system. After shaking vigorously, the contents were mixed thoroughly. Then the equilibration was established and the two phases were analyzed separately by HPLC. The peak area of the target compound in the upper phase was recorded as A_U_, and that in the lower phase was recorded as A_L_. The K value was calculated according to the equation K = A_U_/A_L_. For complex compounds that had two or more peaks, the separation factor (α = K2/K1, where K2 > K1) between them was also calculated.

### 2.5. HSCCC separation procedure

In this study, the two-phase solvent system containing *n*-hexane–ethyl acetatemethanol–water (3:5:4:5 v/v/v/v) was selected for HSCCC separation. The solvent mixture was thoroughly shaken in a separation funnel and the two phases separated quickly. After setting overnight, the two phases were separated into two bottles, and an acidic modifier (0.1% trifluoroacetic acid, TFA) was added to the lower phase. The total volume of the two-phase solvent system was 1,020 mL, and the volume of the upper and lower phases was approximately 400 mL and 620 mL, respectively. The two phases were then treated with an ultrasonic cleaner to remove the bubbles. The 100 mg HSAF crude sample was dissolved in a 10 mL solvent mixture consisting of equal volumes of both the upper and lower phases, and the sample solution was ready for loading.

In the HSCCC procedure, the upper phase acted as the stationary phase and was pumped into the coiled column at a flow rate of 20 mL/min. After the column filled with the stationary phase, the lower phase acted as the mobile phase and was pumped into the column at a flow rate of 2.0 mL/min with the head-to-tail elution model, and at the same time, the apparatus was rotated at 1000 rpm and the temperature was kept at 25°C. After the mobile phase was seen flowing out, a hydrodynamic equilibrium was established. Then, 10 mL of the sample solution was injected into the system. The effluent was monitored with a UV detector at 318 nm and the fractions were manually collected according to the profiles of the chromatographic peaks. The target fractions were evaporated using an electric fan under the air stream to partially remove the organic solvent. The obtained precipitate was collected and evaporated using the rotary evaporator at a water-bath temperature of 50°C under reduced pressure, and the remaining solvents including water and TFA were removed. The remaining products were dissolved in methanol for HPLC analysis.

### 2.6. HPLC analysis for the isolated compound

Qualitative and quantitative analysis of HSAF in the crude extract or in the HSCCC fraction was performed using the Shimadzu LC-20A HPLC system with an isocratic elution mode. The mobile phase was water–acetonitrile (45:55, both containing 0.04% TFA, v/v) at a total flow rate of 1.0 mL/min. The effluent was monitored by a UV detector at 318 nm. The pure standard of HSAF was obtained using the method of macroporous resin adsorption/desorption combined with preparative HPLC as in the previous study (Tang et al., 2018a,b; Tang et al., 2021). The standard compound was diluted into 5, 10, 50, 100, 200, 400, and 500 μg/mL with methanol and injected into HPLC to establish the linear regression equation. The concentration of HSAF in the analyzed solution was quantitatively determined by linear regression using the equation Y = 30,885X − 29,077 (R^2^ = 0.9920), where X is the concentration of HSAF (μg/mL) and Y is the absorption peak area of HSAF. The purity of HSAF was calculated as (the weight of HSAF/the weight of sample) × 100%, and the recovery rate of HSAF in the HSCCC procedure was calculated as (the weight of HSAF obtained after HSCCC/the weight of HSAF before HSCCC) × 100%.

### 2.7. Identification of the isolated compound

The chemical structure of the isolated compound was confirmed by HR-TOF-MS, ^1^H NMR, and ^13^C NMR. The measured spectroscopic data were compared with the reported spectroscopic data (Graupner et al., 1997; Li et al., 2012). In addition, the antifungal activity of the isolated compound was determined using the methods described in our previous studies (Zhao et al., 2019; Li et al., 2021). Briefly, the compound was diluted with DMSO to create different gradient solutions and 0.5 mL of each solution was added into 100 mL of half-melted (approximately 45°C) PDA medium to yield a final concentration of 0.1, 0.2, 0.5, 1, 2, and 5 μg/mL. The same volume of DMSO was added as the control. Then, the center of the solidified medium was inoculated with a 5 mm mycelial plug that was cut from the edge of a fresh fungal colony. The plates were incubated at 28°C for 5 days and there were three replicates in the experiment. The inhibition rates for the gradient solutions were calculated, and the EC_50_ values (the concentration inhibiting 50% of mycelial growth) were calculated using probit-log analysis. Three independent experiments were conducted for the bioactivity assays, and the EC_50_ value was expressed as “mean ± standard deviation.” As a control HSAF, the pure standard compound of HSAF was prepared by prep-HPLC in our laboratory (Tang et al., 2021), and it was tested against the same five plant pathogens.

## 3. Results and discussion

### 3.1. Pretreatment of HSAF crude extract

The biocontrol bacterial strain *Lysobacter enzymogenes* OH11 (CGMCC No. 1978) was demonstrated to produce a high yield of HSAF in our laboratory (Tang et al., 2018a,b; Zhao et al., 2021). In this study, *L. enzymogenes* OH11 strain was inoculated in the defined SGC medium and cultured for 60 h, and then HSAF was effectively adsorbed onto the macroporous adsorption resin NKA. The resin was washed with ethanol to obtain the initial crude extract. After evaporating to dryness, the initial crude extract was obtained as a dark brown gum, and the purity of HSAF in the crude extract was approximately 28.6%. As a result, there were many analogous compounds and impurities in the crude extract. In particular, many compounds were analogous to HSAF such as the main interferent alteramide B (ATB) (Figure 1) (Li et al., 2012; Xu et al., 2015; Ding et al., 2016a,b), and both HSAF and ATB have similar structures and close retention times in HPLC. In the preliminary experiment, the two compounds were usually difficult to separate using prep-HPLC or HSCCC. In order to obtain a better resolution for HSAF in the following procedure, pre-treatment for the initial crude extract was necessary.

**Figure 2:**
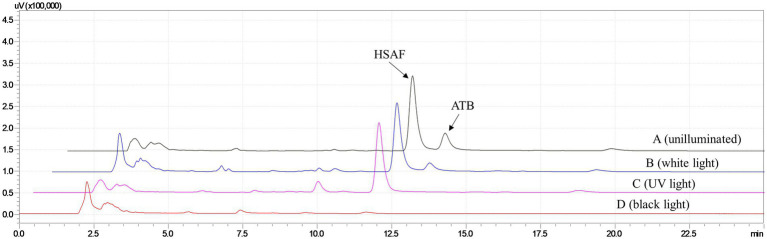
HPLC analysis for the HSAF crude extract from *Lysobacter enzymogenes* OH11 after being illuminated with different light sources for 2 days. (A) unilluminated sample; (B) white light (wavelength at 400–760 nm); (C) ultraviolet germicidal light (wavelength at 254 nm); and (D) black light (wavelength at 365 nm). Peaks of HSAF and ATB are marked in the chromatogram.

In our previous research (Tang et al., 2021), we found that after exposing the fermentation broth to high-intensity fluorescent light for 2 days, most of the ATB was degraded, which could help acquire high purity of HSAF. However, this illumination time was slightly longer, and the effect of different kinds of light on the ATB degradation was unknown. In this study, the ethanol desorption solutions eluted from resin NKA were illuminated with three different light sources, including white light (wavelength at 400–760 nm), ultraviolet germicidal light (wavelength at 254 nm), and black light (wavelength at 365 nm). Furthermore, the ethanol solutions were analyzed by HPLC. The results in Figure 2 show that, after being illuminated with white light for 2 days, the peaks of HSAF and ATB in the ethanol solution were almost the same as those in the unilluminated solution. While under ultraviolet light at 254 nm, the peak of ATB disappeared and HSAF was still present. When exposed to black light at 365 nm, both the ATB and HSAF peaks disappeared. These results showed that ultraviolet light (254 nm) was more effective than white light in eliminating the interference of ATB. Moreover, black light at 365 nm was harmful to both ATB and HSAF. This may be a result of the 5/5/6-tricyclic system being destroyed under black light, as this system is critical to the stability of polycyclic tetramate macrolactam compounds (Huffman et al., 2010; Tang et al., 2021).

**Figure 3:**
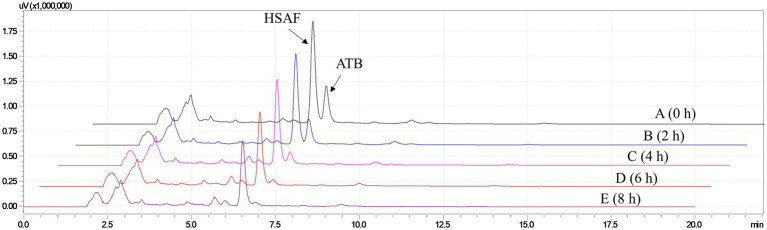
HPLC analysis for the HSAF crude extract illuminated with ultraviolet germicidal light at different times. (A) 0 h; (B) 2 h; (C) 4 h; (D) 6 h; and (E) 8 h. Peaks of HSAF and ATB are marked in the chromatogram.

Furthermore, different times of ultraviolet light exposure were evaluated, and the results are shown in Figure 3. As can be seen from the HPLC results, the peak of ATB decreased with the illumination time setting from 0 h to 8 h, and we found that an illumination time of 6 h was enough for the ATB degradation. Based on these results, illumination with ultraviolet light for 6 h was proposed for eliminating the interference of ATB in the pretreatment of crude extract. According to the peak area measured by HPLC, the content of HSAF decreased by approximately 15.7% after exposure to ultraviolet light. Compared with our previous research, the illumination time was shortened from 2 days to 6 h.

In order to remove the impurities that could not be detected by the HPLC UV detector, column chromatography on Sephadex LH-20 was necessary. Using an organic phase (such as dichloromethane-methanol = 1:1 herein) as the isocratic elution solvent on the Sephadex LH-20 column can prevent the irreversible adsorption of the samples, and this method has been widely used for the separation and purification of natural products (Mottaghipisheh and Iriti, 2020). In this study, after illumination with ultraviolet light for 6 h, the HSAF extract was repeatedly loaded on the column using dichloromethane-methanol = 1:1 as the elution solvent. The effluent fractions containing HSAF were combined and concentrated. After pretreatment of the crude extract with UV light and column chromatography, the purity of HSAF in the extract reached approximately 45.8%, and this was favorable to the next HSCCC procedure.

### 3.2. Optimization of HSCCC separation

#### 3.2.1. Selection of two-phase solvent system

The selection of a suitable two-phase solvent system is crucial for a successful separation by HSCCC. In a previous review (Ito, 2005), Ito summarized that the solvent system should satisfy the following requirements: (1) the partition coefficient “K values” for the target compounds should fall between 0.5 and 2, (2) the separation factor “α” for the two compounds (α = K2/K1, where K2 > K1) should be greater than 1.5, and (3) the settling time of the two-phase solvent system should be <20 s because it is important for the retention of the stationary phase and higher retention of the stationary phase gives better peak resolution.

Considering that HSAF is partly soluble in ethyl acetate and methanol but hardly soluble in alkane and water, in the present study, we selected the widely used solvent system composed of “*n*-hexane/ethyl acetate/methanol/water” to calculate the K values. The HSAF sample solutions mixed in different two-phase solvents were prepared, and the K values for HSAF and ATB were calculated based on the peak area from HPLC. The results are summarized in Table 1. As shown in Table 1, when the ratios of *n*-hexane or methanol were high in the upper or lower phase (system No. of 1–3), the K values of HSAF were below 1.0, which indicated that the compound was mostly distributed in the lower aqueous phase. As the ratios of *n*-hexane or methanol decreased (system No. 4–6), the K values of HSAF became larger than 2.0, indicating that the compound mostly moved into the upper organic phase. Finally, the solvent system composed of *n*-hexane/ethyl acetate/methanol/water (3:5:4:5, v/v) (system No. 7) was selected as it gave a suitable K value of 1.29 for HSAF. However, we found that the K value of ATB was similar to that of HSAF in the different solvents, and a higher K value of 2.02 was observed for ATB in the selected solvent system, indicating that ATB may have a later retention time than HSAF in HSCCC. The separation factor (α = 1.57) between ATB and HSAF was slightly larger than 1.5, and this may be unfavorable for the ideal HSCCC separation. However, considering that the interferent ATB could be degraded with the help of UV light, the solvent system No. 7 could be considered to be used in further separation.

**Table 1:** The partition coefficients (K Values) and separation factors (α) for HSAF and ATB in different solvent systems.

No.	Solvent system	K value (HSAF)	K value (ATB)	Separation factor (α)
1	*n*-hexane/ethyl acetate/methanol/water 6:4:5:5	0.07	0.09	1.29
2	*n*-hexane/ethyl acetate/methanol/water 5:5:5:5	0.20	0.26	1.30
3	*n*-hexane/ethyl acetate/methanol/water 4:5:4:5	0.56	0.84	1.50
4	*n*-hexane/ethyl acetate/methanol/water 3:5:3:5	3.10	5.94	1.92
5	*n*-hexane/ethyl acetate/methanol/water 2:5:2:5	17.01	35.52	2.09
6	*n*-hexane/ethyl acetate/methanol/water 1:5:1:5	93.49	171.59	1.84
7	***n*-hexane/ethyl acetate/methanol/water 3:5:4:5**	**1.29**	**2.02**	**1.57**

The bold values mean the selected optimal values in the experiment.

More importantly, organic acids (acidic modifiers) are always added to the solvent system to improve the resolution of compounds. In this study, we found that adding 0.1% TFA into the lower phase gave a better separation for HSAF from other impurities, especially when the sample size increased. This may be a result of the HSAF molecules becoming more hydrophobic and favoring partition in the organic phase due to protonation (Ito, 2005), and adding acid could help push the equilibria of multiple tautomers of HSAF into a single isomer so that the chromatographic behavior would work better. Thus, 0.1% TFA was added to the lower aqueous phase in the following experiment.

#### 3.2.2. Flow rate of mobile phase

The flow rate of the mobile phase has a considerable impact on separation time, retention of the stationary phase, and peak resolution in the HSCCC (Ito, 2005; Kang et al., 2016). A lower flow rate may give a higher retention of the stationary phase and a better resolution, but it may take a longer time to separate. In this study, different flow rates of the mobile phase were investigated using the same solvent system to separate HSAF from the crude extract. The results in Table 2 show that, when the flow rate increased from 1.5 mL/min to 3.0 mL/min, the retention time of HSAF was shortened from 150 min to 80 min, and the retention of the stationary phase gradually decreased from 59.4 to 34.4%. Higher retention of the stationary phase gives a higher resolution for the compounds, and too low retention is not acceptable. Considering that the partitioning of HSAF could be more efficient in the fixed 1.6 mm i.d. PTFE tubing with a 300 mL column, a lower flow rate was adopted. Thus, a flow rate of 2.0 mL/min was selected as the preferable value due to its reasonable retention of the stationary phase (43.8%) and acceptable retention times for HSAF (115 min).

**Table 2:** Effect of mobile phase flow rate on the retention time of HSAF and the retention of the stationary phase in HSCCC.

Flow rate of mobile phase	Retention time of HSAF	Retention of stationary phase
1.5 mL/min	150 min	59.4%
**2.0 mL/min**	**115 min**	**43.8%**
2.5 mL/min	95 min	37.5%
3.0 mL/min	80 min	34.4%

The bold values mean the selected optimal values in the experiment.

#### 3.2.3. Sample size

As a semi-preparative or preparative separation technique, one of the most important goals for HSCCC is to obtain as many products as possible in a single run under specific conditions (Harada et al., 2001); however, the sample size is limited by certain HSCCC instruments and the selected separation conditions. The resolution between the peaks is the main factor that limits sample size (Zhao and He, 2007; Ren et al., 2013). As the sample size increased, the target compound more easily tended to flow out with the impurities at the early retention time, and the eluted bands of the target compound would become broader than normal. Furthermore, the stationary phase was easily lost.

In this study, the sample size of the HSAF crude extract was gradually increased from 50 mg to 200 mg, which was dissolved in 10 mL of equal volumes of both upper and lower phases, and injected into the HSCCC system. We found that the peak height of HSAF gradually increased as the sample size increased, and the eluted bands became broader. However, when the sample size increased to 150 mg, a large amount of HSAF easily flowed out along with the impurities during the first 90 min, and later, the amount of the remaining HSAF fraction (120 min) was lower than before. So most of the target compound was lost due to the separation capacity being limited by the current conditions. In order to retain a satisfactory resolution, 100 mg of HSAF crude extract was selected as the sample size for semi-preparative HSCCC. These results remind us that if we want to obtain more product, we can consider using a preparative HSCCC instrument with a column volume at the “liters” level as an alternative method, which would allow the sample to be separated at the “gram” scale.

#### 3.2.4. HPLC analysis and treatment of HSCCC fractions

**Figure 4:**
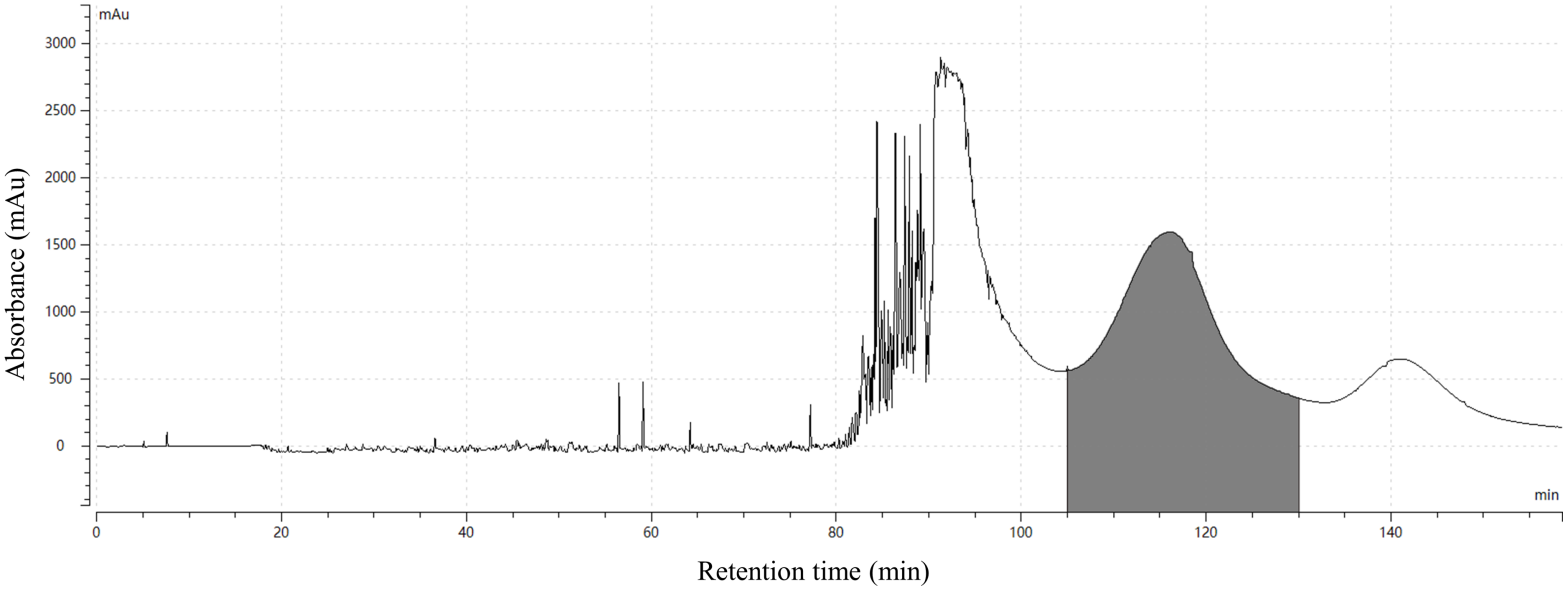
HSCCC chromatogram for the separation of HSAF from the crude extract. The solvent system was composed of *n*-hexane/ethyl acetate/methanol/water (3:5:4:5, v/v, the lower phase added with 0.1% TFA); the upper phase was used as the stationary phase, and the lower phase was used as the mobile phase; flow rate of the mobile phase was 2.0 mL/min; the rotation speed was 1,000 rpm; the UV detection wavelength was at 318 nm; the separation temperature was 25°C; the retention in the stationary phase was 43.8%; and the loading sample size was 100 mg. The fractions containing HSAF were collected at 105 ~ 130 min (shaded area in the map).

**Figure 5:**
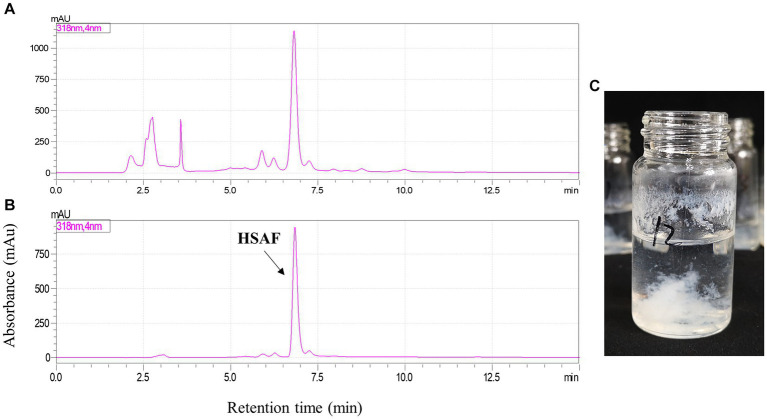
HPLC analysis of HSAF sample before and after HSCCC separation. **(A)** HSAF crude extract before HSCCC separation; **(B)** HSAF fraction collected at 105 ~ 130 min; and **(C)** The pure HSAF white floccule formed in the glass bottle.

Finally, the separation of 100 mg HSAF crude extract under the selected operating conditions (1,000 rpm revolution speed, 2.0 mL/min flow rate, and 25°C temperature) was performed using *n*-hexane/ethyl acetate/methanol/water (3:5:4:5, v/v, and the lower phase was modified with 0.1% TFA) as the solvent system in HSCCC. The eluted fractions were collected manually according to the chromatographic profile and then analyzed by HPLC. The typical HSCCC chromatogram is shown in Figure 4. The result showed that HSAF had well separated from other impurities within the 160 min running time. Most of the impurities flowed out at the front of 100 min, and no HSAF appeared. At about 105 min, HSAF started to flow out, and a broad peak was formed until 130 min. The fraction was collected and analyzed by HPLC, and the results are shown in Figures 5A,B. It was shown that the peak of HSAF was more prominent after being separated by HSCCC. In order to facilitate the precipitation of the compound in the aqueous solution, the fraction was treated with an electric fan at room temperature.

The volatile organic solvents in the HSCCC fraction collected at 105 ~ 130 min were removed using an electric fan working overnight. After one night, a mass of white floccule was formed in the glass bottle (Figure 5C), and it was separated by centrifugation. The precipitate was dried, weighed, and dissolved in methanol. In the quantitative analysis by HPLC, the purity of HSAF in the white floccule was approximately 97.6%, indicating that the white floccule was high purity of HSAF. The weight of high-purity HSAF obtained in the HSCCC was 42 mg, and the recovery rate of HASF in the loading sample was determined as 91.7%.

HSAF is a novel antifungal active compound that has broad application prospects in both agriculture and medicine. This compound has attracted persistent attention from our research team. Previous studies had developed many strategies for separating and enriching pure HSAF from *L. enzymogenes*. In the ethyl acetate extraction method, the purity of HSAF in the brown paste product was only 8.67% (Tang et al., 2021). In the NKA resin adsorption method, the HSAF content in the light yellow product reached 31.07% (Tang et al., 2021). In the preparative HPLC method, the purity of HSAF could reach as high as 96.54% (Tang et al., 2021). Recently, a method combined with microporous resin and prep-HPLC was developed for isolating HSAF, where the purity and recovery of HSAF in the microporous resin eluent were 68.46 and 70.55%, respectively, and the purity and recovery of HSAF in the prep-HPLC were 95.3 and 52.48%, respectively (Zhong et al., 2022). Compared with the methods mentioned above, the HSCCC method developed in the present study leads to both higher purity (97.6%) and a higher recovery (91.7%) of products, pointing to important progress in enriching pure HSAF. In addition, although prep-HPLC could also yield high-purity HSAF, the price costs for the instrument, columns, and solvents in prep-HPLC were much higher than that for HSCCC, and this is unacceptable in industrial production. In short, the HSCCC method is helpful for obtaining large amounts of pure HSAF at a low cost, and the high recovery rate will also be beneficial for industrial isolation of HSAF derived from *L. enzymogenes* fermentation, as the production efficiency in the factory is always considered.

In a previous study, Harada et al. (2001) used high-speed counter-current chromatography to separate WAP-8294A components from the fermentation broth of *Lysobacter* sp. As WAP-8294A is soluble in water and not readily soluble in organic solvents, a hydrophilic two-phase solvent system composed of *n*-butanol–ethyl acetate–aqueous 0.005 M TFA (1.25:3.75:5, v/v/v) was selected. The separation was performed at a low flow rate of 0.5 mL/min and the separation time was as long as 13.3 h. The retention of the stationary phase was measured as 45.2%. The sample size was 25 mg, the yielded pure fractions for three components were 1 ~ 6 mg, and the total recovery was 85%. Compared with Harada’s study, our study showed some similar features, in that we all added an organic acid (TFA) to the solvent system to protonate the molecule so that the target compounds were mostly partitioned into the organic phase. In the separation of HSAF, this study showed more competitive advantages as the separation time was much shorter, the sample size was much bigger, and the recovery was much higher. However, more research work needs to be done to improve the performance of HSCCC in developing natural compounds from *Lysobacter* sp., such as using HSCCC as the tool to isolate new compounds, increase the yields of pure compounds in one separation, and expand its application in the pharmaceutical industry. The present study provided different perspectives to facilitate the discovery of deep learning in *Lysobacter* sp. and presented novel directions that deserve to be further explored.

### 3.3. Identification of the isolated compound

#### 3.3.1. Structure elucidation

In order to confirm the isolated compound was HSAF, the structure of the compound was elucidated. The isolated compound was dried as a white amorphous powder and dissolved in DMSO-*d*_6_. Then, its chemical structure was measured using HR-TOF-MS, ^1^H NMR, and ^13^C NMR (see Supplementary material). The measured NMR chemical shifts were compared with the data from HSAF or 3-deOH-HSAF from previous studies (Graupner et al., 1997; Li et al., 2012). In the HR-MS spectrum, this compound exhibited a prominent pseudomolecular ion peak at m/z 513.2965 [M + H]^+^ (calculated for C_29_H_41_N_2_O_6_, 513.2959) with 11 degrees of unsaturation (Supplementary Figure S1). The UV spectrum showed maximum absorptions at 219 and 322 nm (Supplementary Figure S2). Its ^1^H NMR and ^13^C NMR shifts were recorded (Supplementary Figures S3, S4). The assigned NMR chemical shifts for the isolated compound and 3-deOH-HSAF in the same solvent of DMSO-*d*_6_ were compared (Supplementary Table S1), and the NMR data for both compounds were very similar, confirming that the isolated compound was HSAF.

#### 3.3.2. Bioactivity assays

**Figure 6:**
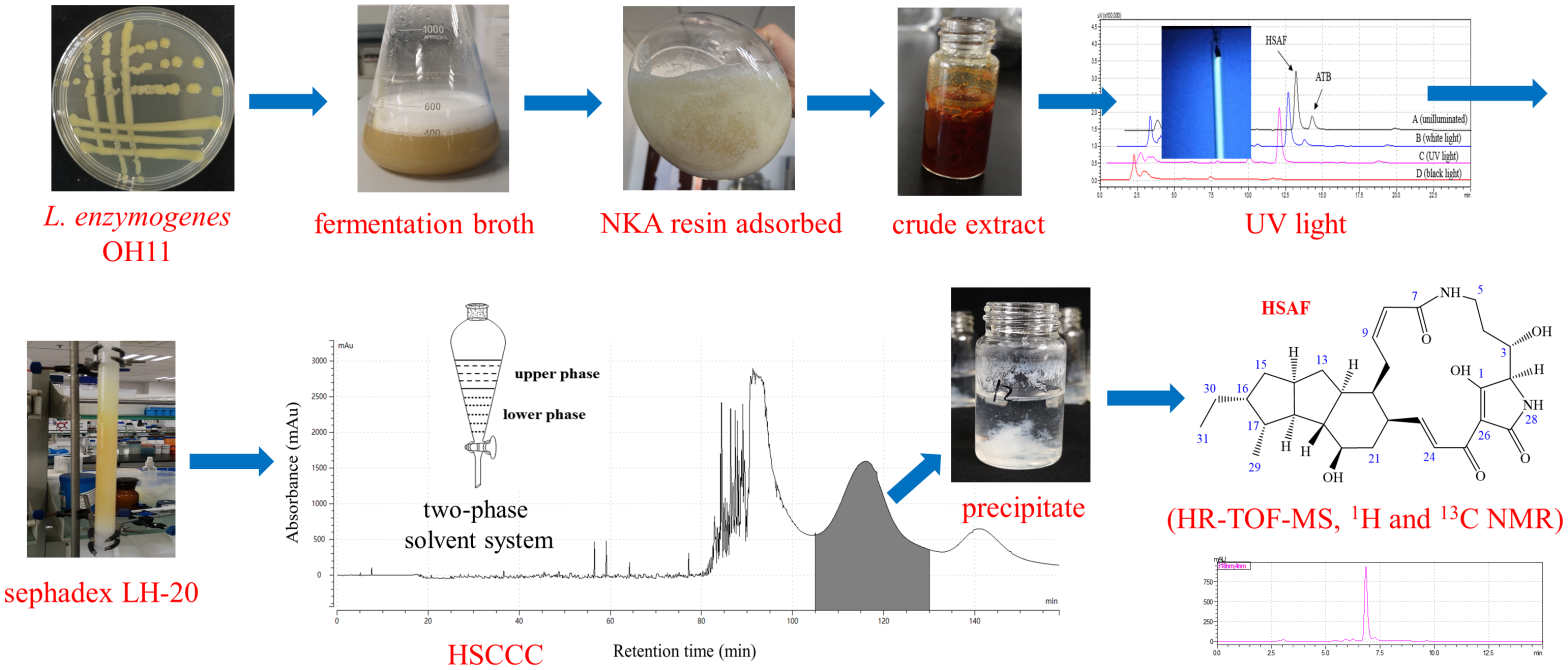
A graphical representation of the separation and purification of HSAF in this study.

To confirm the bioactivity of the isolated compound, the antifungal activity was tested against five plant pathogens, including *Fusarium graminearum* (pathogen of Fusarium head blight), *Colletotrichum fructicola* (pathogen of pear anthracnose), *Alternaria alternata* (pathogen of pear black spot disease), *Botryosphaeria dothidea* (pathogen of pear ring rot disease), and *Valsa pyri* (pathogen of pear canker disease). As shown in Table 3, the isolated compound showed strong antifungal activities against the target fungi, with EC_50_ values ranging from 0.46 to 1.22 μg/mL. Meanwhile, EC_50_ values for the control HSAF ranged from 0.43 to 1.23 μg/mL, and it showed a high degree of similarity with the value of the isolated compound. Thus, HSAF has the potential to be widely applied for the suppression of plant fungal diseases. Some representative images showing the antifungal activity of the isolated compound are shown in Supplementary Figure S5. Based on the structure elucidation and bioactivity assay results, the isolated compound from HSCCC was unambiguously identified as HSAF. The whole procedure for the separation and preparation of HSAF in this study is illustrated in Figure 6.

**Table 3:** Antifungal activities of the isolated compound in this study and control substance of HSAF.

Pathogenic fungi	Isolated compound in this study			Control substance of HSAF		
	Regression equation	Correlation coefficient (*R*^2^)	EC_50_ (μg/mL)	Regression equation	Correlation coefficient (*R*^2^)	EC_50_ (μg/mL)
*Fusarium graminearum*	y = 1.1674x + 5.3131	0.9887	0.54 ± 0.02	y = 1.2769x + 5.3575	0.9867	0.51 ± 0.04
*Colletotrichum fructicola*	y = 1.222x + 5.2053	0.9889	0.69 ± 0.03	y = 1.2646x + 5.2347	0.9850	0.67 ± 0.04
*Alternaria alternata*	y = 1.0952x + 4.9099	0.9959	1.22 ± 0.04	y = 1.1964x + 4.8976	0.9881	1.23 ± 0.07
*Botryosphaeria dothidea*	y = 1.287x + 5.4355	0.9895	0.46 ± 0.03	y = 1.2189x + 5.4397	0.9858	0.43 ± 0.04
*Valsa pyri*	y = 1.0172x + 5.0729	0.9964	0.83 ± 0.06	y = 1.1782x + 5.0901	0.9799	0.86 ± 0.04

## 4. Conclusion

In summary, high-speed counter-current chromatography (HSCCC) was successfully applied for the separation and preparation of HSAF from the extract of *L. enzymogenes* OH11. A suitable two-phase solvent system (*n*-hexane/ethyl acetate/methanol/water = 3:5:4:5, the lower phase added with 0.1% TFA), a flow rate of 2.0 mL/min, and a sample size of 100 mg were selected for the method. The purity of HSAF could reach 97.6% and the recovery was 91.7%. The chemical structure and antifungal activities of the isolated compound were reconfirmed. Compared with conventional methods, HSCCC showed both higher purity and a higher recovery rate, demonstrating that HSCCC is a powerful tool for isolating natural products.

## Data availability statement

The original contributions presented in the study are included in the article/Supplementary material, further inquiries can be directed to the corresponding author.

## Author contributions

WS, JX, and FL conceived and designed the experiments. WS and LD performed the experiments. WS, BT, and YZ analyzed the data. WS wrote the manuscript. YZ and FL modified the manuscript. All authors contributed to the article and approved the submitted version.

## Funding

This work was funded by the Jiangsu Agricultural Science and Technology Innovation Fund [CX(21)3092], the National Key Research and Development Program (2021YFD1400200), and the Earmarked Fund for China Agriculture Research System (CARS-28).

## Conflict of interest

The authors declare that the research was conducted in the absence of any commercial or financial relationships that could be construed as a potential conflict of interest.

## Publisher’s note

All claims expressed in this article are solely those of the authors and do not necessarily represent those of their affiliated organizations, or those of the publisher, the editors and the reviewers. Any product that may be evaluated in this article, or claim that may be made by its manufacturer, is not guaranteed or endorsed by the publisher.
