# Supplementary material for: A novel and high-efficient method for the preparation of heat-stable antifungal factor from Lysobacter enzymogenes by high-speed counter-current chromatography
Source: Front Microbiol. 2023 Aug 14;14:1227244. doi: 10.3389/fmicb.2023.1227244 (PMC10461446; doi:10.3389/fmicb.2023.1227244)
Supplement: Supplementary file 1 [file Data_Sheet_1.docx]

Supplementary Material


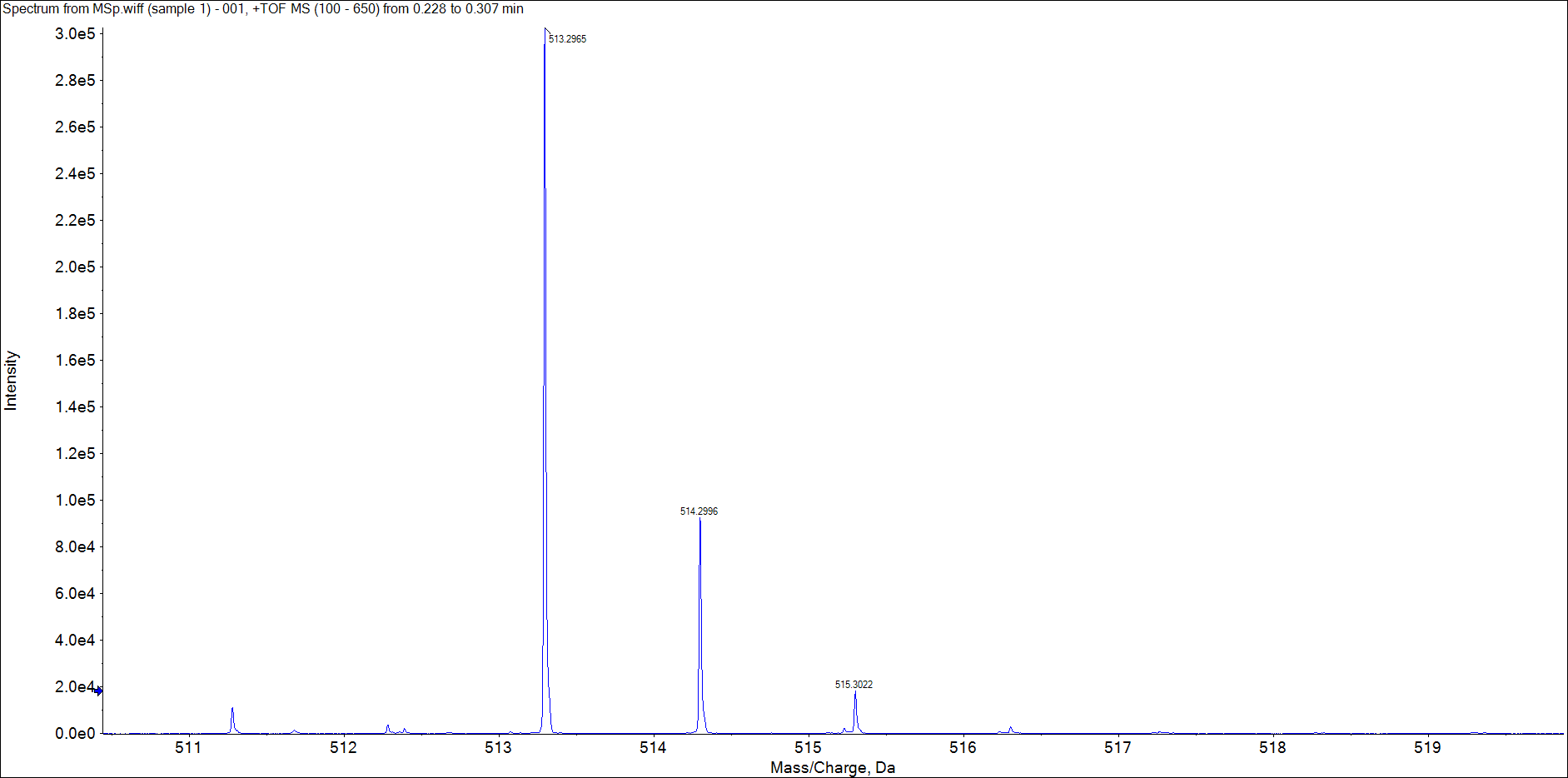


513.2965

**Fig. S1.** HR-TOF-MS spectrum of the isolated compound (pseudomolecular ion peak at m/z 513.2965 [M+H]).

**Fig. S2.** UV spectrum of the isolated compound.


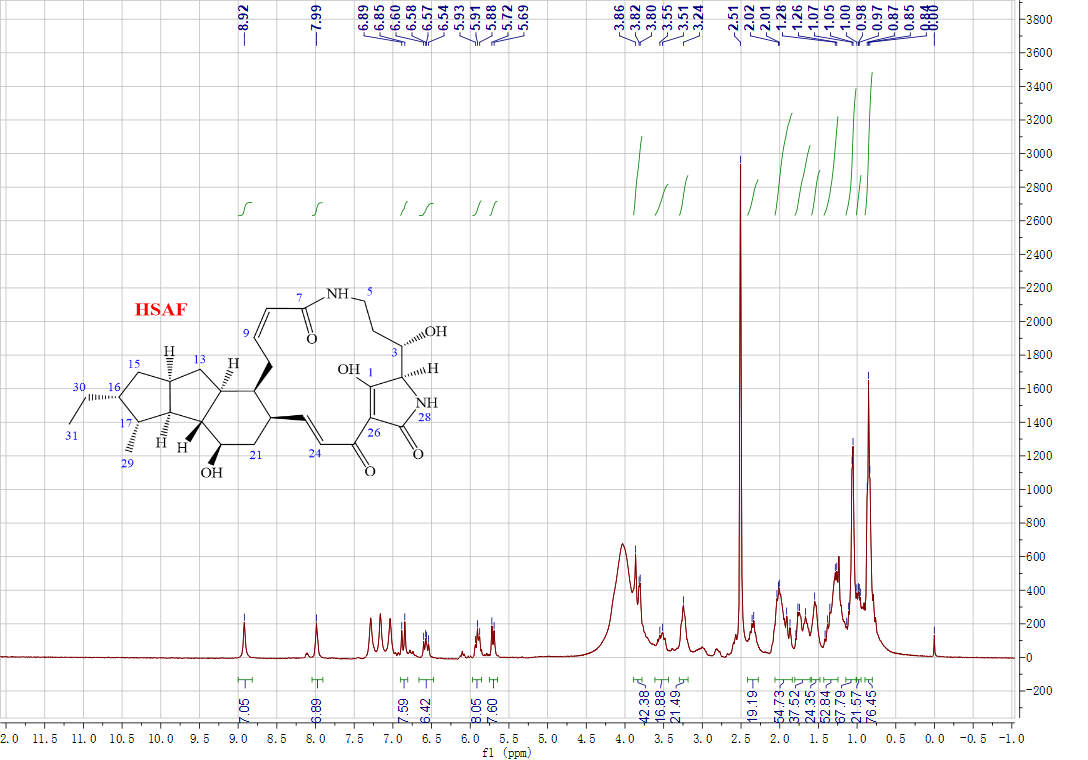


**Fig. S3.** ^1^H NMR spectrum of the isolated compound (DMSO-*d*_6_, 400MHz).


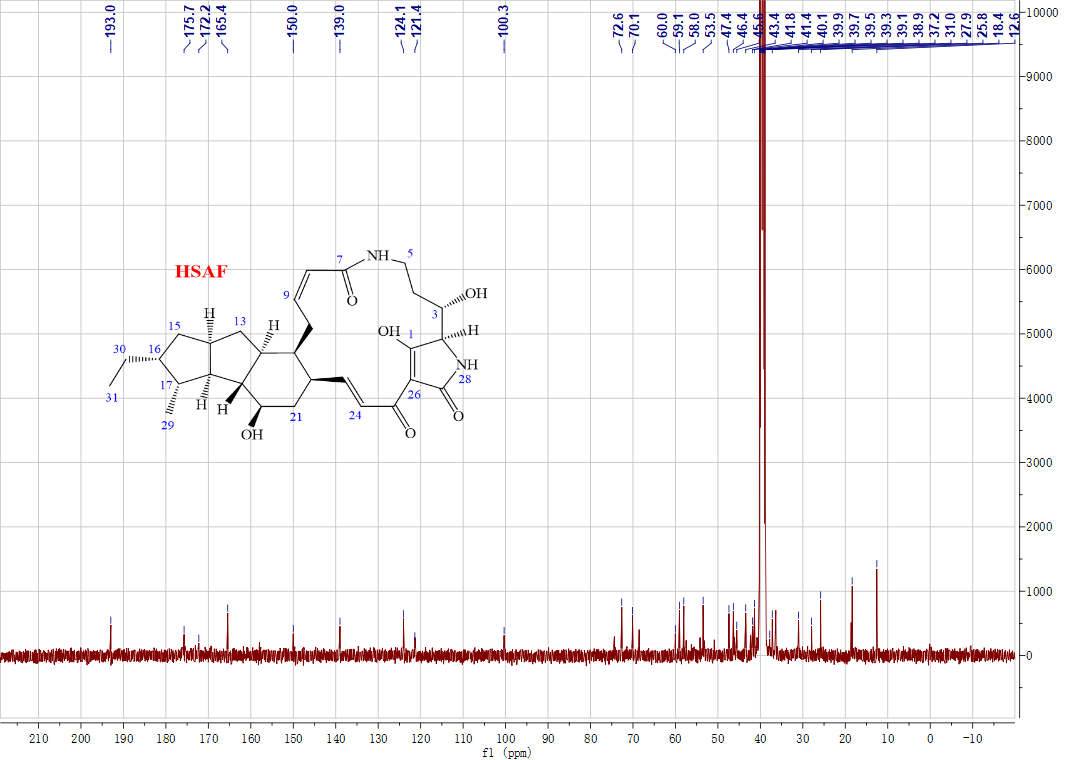


**Fig. S4.** ^13^C NMR spectrum of the isolated compound (DMSO-*d*_6_, 100MHz).


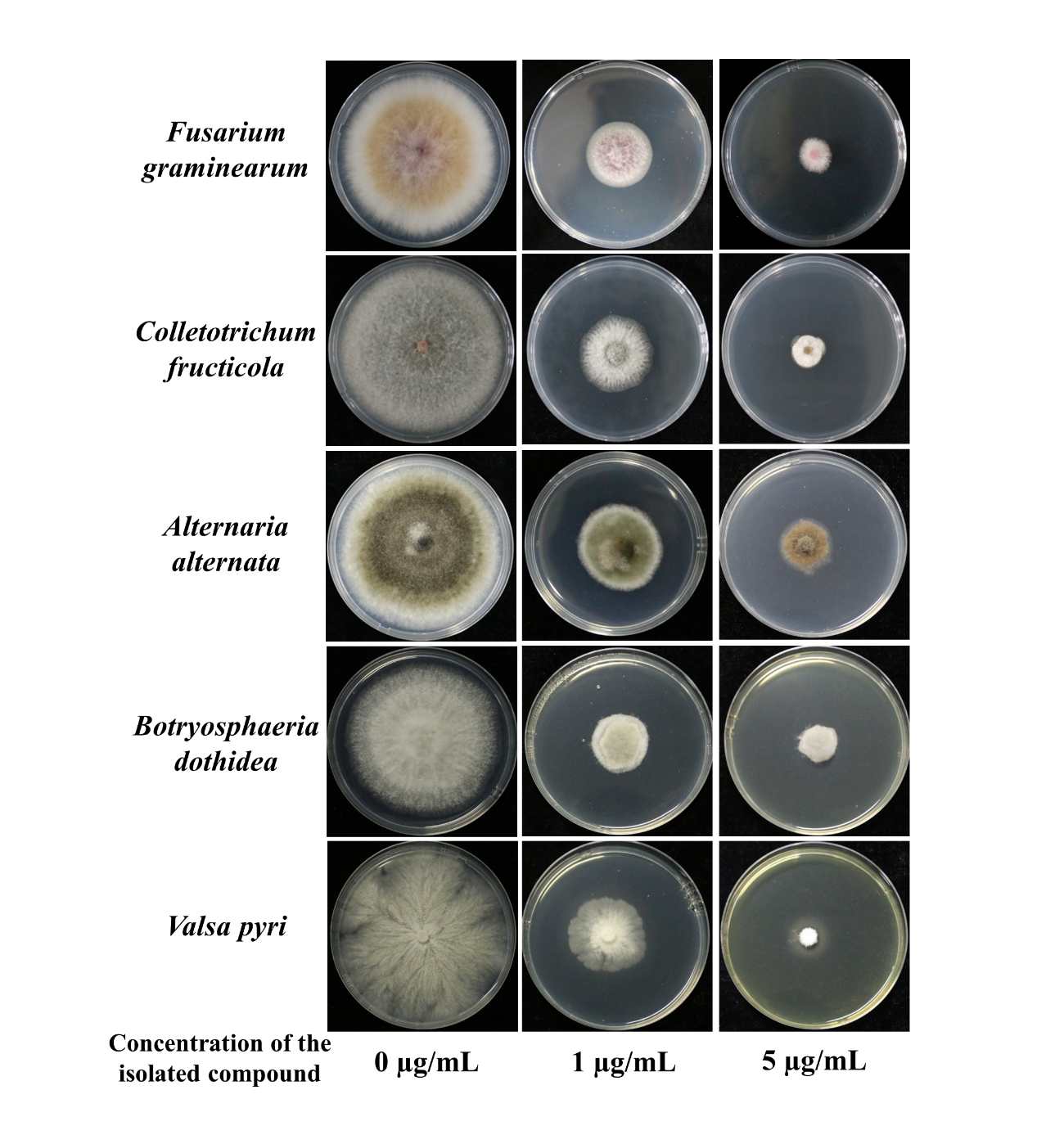


**Fig. S5.** Antifungal activity of the isolated compound.

**Table S1.** Assigned chemical shifts of the NMR spectra for the compound and the comparison with the reference data.

| Position | Measured data  (DMSO-*d*_6_) | | Reference data [1]^a^  (DMSO-*d*_6_) | |
| --- | --- | --- | --- | --- |
|  | **δ (H)**  **(multiplicity, J in Hz)** | **δ (C)**  **(type)** | **δ (H)**  **(multiplicity, J in Hz)** | **δ (C)**  **(type)** |
| 1 |  | 193.0 (C) |  | 195.7 (C) |
| 2 | 3.81 (d, 5.6) | 60.0 (CH) | 3.82 (d, 5.3) | 61.0 (CH) |
| 3 | 1.55 (m), 1.06 (m) | 70.1 (CH) | 1.57 (m), 1.06 (m) | 25.8 (CH_2_) |
| 4 | 1.38 (m), 1.13 (m) | 31.0 (CH_2_) | 1.40 (m), 1.15 (m) | 20.4 (CH_2_) |
| 5 | 3.24 (m), 2.36 (m) | 37.8 (CH_2_) | 3.26 (m), 2.40 (m) | 38.0 (CH_2_) |
| 6 | 7.99 (s) |  | 7.84 (t, 5.3) |  |
| 7 |  | 165.4 (C) |  | 165.5 (C) |
| 8 | 5.71 (d, 11.6) | 124.1 (CH) | 5.74 (d, 11.0) | 124.1 (CH) |
| 9 | 5.91 (t, 10.8) | 139.0 (CH) | 5.92 (td, 11.0, 1.9) | 138.9 (CH) |
| 10 | 3.53 (d, 14.2), 1.91 (m) | 27.9 (CH_2_) | 3.52 (t, 12.1), 1.93 (d, 16.7) | 28.0 (CH_2_) |
| 11 | 1.28 (m) | 46.4 (CH) | 1.36 (m) | 46.3 (CH) |
| 12 | 1.67 (m) | 47.4 (CH) | 1.67 (m) | 47.5 (CH) |
| 13 | 2.01 (m), 0.85 (m) | n.d. | 2.03 (m), 0.81 (m) | 40.2 (CH_2_) |
| 14 | 2.34 (m) | 41.4 (CH) | 2.37 (m) | 41.3 (CH) |
| 15 | 2.02 (m), 0.98 (m) | 37.2 (CH_2_) | 2.03 (m), 0.92 (m) | 37.2 (CH_2_) |
| 16 | 1.41 (m) | 53.5 (CH) | 1.41 (m) | 53.4 (CH) |
| 17 | 1.35 (m) | 43.4 (CH) | 1.37 (m) | 43.4 (CH) |
| 18 | 1.74 (m) | 58.0 (CH) | 1.74 (m) | 58.0 (CH) |
| 19 | 1.11 (m) | 59.1 (CH) | 1.12 (m) | 59.0 (CH) |
| 20 | 3.24 (m) | 72.6 (CH) | 3.27 (m) | 72.6 (CH) |
| 21 | 1.76 (m), 1.26 (m) | 41.8 (CH_2_) | 1.76 (m), 1.35 (m) | 41.8 (CH_2_) |
| 22 | 2.04 (m) | 45.6 (CH) | 2.11 (qd, 11.3, 2.8) | 45.6 (CH) |
| 23 | 6.57 (dd, 14.4, 10.7) | 150.0 (CH) | 6.58 (dd, 15.4, 10.6) | 149.6 (CH) |
| 24 | 6.87 (d, 15.6) | 121.4 (CH) | 6.95 (d, 15.4) | 121.4 (CH) |
| 25 |  | 175.7 (C) |  | 175.2 (C) |
| 26 |  | 100.3 (C) |  | 100.6 (C) |
| 27 |  | 172.2 (C) |  | 171.6 (C) |
| 28 | 8.92 (s) |  | 8.70 (d) |  |
| 29 | 1.06 (d, 5.1) | 18.4 (CH_3_) | 1.06 (d, 6.2) | 18.4 (CH_3_) |
| 30 | 1.87 (m), 1.79 (m) | 25.8 (CH_2_) | 1.87 (m), 1.78 (m) | 26.2 (CH_2_) |
| 31 | 0.85 (t, 6.8) | 12.6 (CH_3_) | 0.86 (t, 7.1) | 12.5 (CH_3_) |

^a^ The δ (H) and δ (C) data were recorded for 3-deOH-HSAF in DMSO-*d*_6_ at 600 MHz and 150 MHz, whose structure was similar with HSAF, except for the absence of the hydroxyl group at C-3.

**Reference**

[1] Y. Li, J. Huffman, Y. Li, L. Du, Y. Shen. 3-Hydroxylation of the polycyclic tetramate macrolactam in the biosynthesis of antifungal HSAF from *Lysobacter enzymogenes* C3, Med. Chem. Commun. 3 (2012) 982, doi:10.1039/c2md20026k.
